# Selections from Foreign Periodicals

**Published:** 1887-07

**Authors:** 


### SELECTIONS FROM FOREIGN PERIODICALS.

Changes Produced in the Lungs of Sheep by a Parasitic Worm, (*Strongylus filaria.*) By Henry Bewley, M. B.—*Journal of Anat. and Phys.*, April.

On several occasions lately some sheep's lungs have been obtained for the Physiological Laboratory in Trinity College, Dublin. Prof. Purser drew my attention to the presence in many of these lungs of small nodules or tubercles, whitish-grey in color, some about the size of a small shot, others larger, being as much as one-eighth inch or more in diameter, In fact, they much resembled the miliary tubercles seen in human lungs in cases of acute tuberculosis. They existed in large numbers, both on the surface of the lung, immediately under the pleura, and also in the deeper parts of the organ. They seemed to be especially numerous along the thin edge of the lung, where they formed small, hard, shot-like elevations. The surrounding lung tissue seemed healthy. From some nodules in a fresh lung, by tearing them up with needles, I obtained some specimens of the entire *Strongylus filaria.* Two of the worms were respectively fifteen and seventeen milimetres long, and very slender, appearing hair-like. I could not find any trace of reproductive organs. There was a curious arrangement of the intestinal canal in the middle part of these worms, which is not like that in any other nematode I know of. The anterior path of the intestine is quite straight; the middle part is coiled into a regular corkscrew-like spiral; the posterior part is again straight. It is common enough for part of the intestine in nematodes to be alternately constricted and dilated; but I have not been able to find any drawing or description of a regular spiral arrangement such as exists in these worms. Leuckart and Baillet allude to the nodules which these worms cause in the lungs, but they give no details of their histological structure. The adult and mature forms, of which these are the embryos, are from 50 to 100 millimetres long, and inhabit the trachea and bronchi of sheep and goats, where they cause bronchitis. The females are viviparous, and give birth to minute worms about six milimeters long. These, having crept or having been coughed out of the animal's mouth, pass, according to Leuckart, into the body of some insect or snail, where they undergo further development. Afterwards they are again taken into the mouth of a sheep, probably with moist grass, and from its mouth or œsophagus creep into its larynx and trachea. In ruminants this can the more readily occur, because the food, having been swallowed, is brought up a second time into the mouth. The life-history of these worms from the time they are taken into the sheep's mouth to that when they are found as full-grown sexual worms in the bronchi, does not appear to be well known. I do not think that the being encysted in the lungs forms any stage of their normal life-history. It seems more probable that they normally remain in the trachea and there develop to full maturity, and that the individuals which are found in the nodules in the lungs have, so to speak, lost their way among the tissues of their host, and that they remain in the nodules till they die. This view I think probable, because: 1. A good many of the nodules contain, not living worms, but the remains apparently of dead worms. 2. All the worms in the various nodules are about the same size, and none appear to be more fully developed than the rest. 3. It is not easy to see how these worms could escape out of such nodules as these, and bore their way through the lung tissue till they arrived at a bronchus of sufficient size to contain them.—*Medical Abstract.*

On the Physiology of the Sebacious Glands. By Dr. Max Joseph, Berlin.—*Centralblatt fur Physiologie* for April, 1887.

The object of the author is to establish the importance, from the standpoint of comparative physiology, of the sebacious glands secretion in the lubrication of the hairs and feathers. He quotes Professor Liebrich's discovery (Berl. Klin. Woch., 1885, No. 47) of the large amount of cholesterine fat in the epidermic cells, and asks whether this intra-cellular fat is absolutely sufficient for the normal preservation of hairs and feathers, or whether the additional fat of of the sebacious glands plays an important part.

To settle this question he determined to operate. It would naturally be impossible, he says, to exterpate the sebacious glands where they are distributed over the whole surface of the body, as is the case in the sucking animals; but, fortunately, we have in birds the breast glands (Bruzel drussen), which are the equivalent of the sebacious glands in sucking animals. These glands are very easily exterpated. In the animals operated upon, healing generally took place by first intention in a few days. There was no functional disturbance and no deaths followed the operation.

His next experiments were to determine what effect water would have upon them under their altered condition. For this purpose, several more ducks were operated upon. In about ten days after the operation they were weighed, then immersed in a large tub of water, and immediately weighed again. The animals were then allowed to move about freely in a large room, to shake off the water from their feathers, and in about a quarter of an hour they were again weighed, this time each animal separately. The same methods were adopted with a number of normal animals taken for control.

The amount of water taken up was about the same in each case, viz: an average of 465 grammes for normal ducks which had been operated upon. On the other hand, however, after a quarter of an hour's shaking off of the water, there remained in the feathers of normal ducks 56.6 grammes, and in the feathers of ducks which had been operated upon 137.7 grammes.

The inference drawn from this experiment is that normal ducks, and those which have been operated upon, take up about the same amount of water, but that the latter retain from two to two and a half times more water in their feathers than do sound animals. Normal ducks, moreover, required but a slight movement to dry themselves, while ducks which had been operated upon, required to shake themselves very energetically, and for a long time, before their feathers were free from water.

The author omits to state his conclusions until he has finished his whole series of experiments.

A. W. Clement, V. S.

Rupture of the Aorta.—Halle (Archiv. fur Wiss. Prakt. Thierheilkunde, Bd. xii., 3 and 4 Heft, 1886) saw a case of rupture of the aorta in the horse, which was sent to him to be treated for what was believed to be the result of severe beating of the animal by the groom. After examining the horse, Halle found, between the thirteenth and fourteenth ribs, about twelve cm. from the spine, a wound, about one cm. in length, discharging a yellowish matter. The wound led in a straight direction to a large saccular termination. To give vent to the contained matter, he freely opened the wound from below. Marked hæmorrhage followed this operation, which could not be checked. The animal died in a few minutes from excessive bleeding. On post mortem examination, he found below each saccular dilatation, an enlargement of the size of a man's head, which was situated more to the left of the above saccule, and was separated from it by the peritoneum only. The enlargement contained a solid, blood-stained substance and completely encircled the posterior aorta; the inner half of the aorta had ruptured nearest to the enlargement. Through the operation on the enlargement, the aorta, which was separated merely by peritoneum, was reached, and hence the bleeding which followed. Further examination of the body revealed two other enlargements near the kidneys, and which were connected with the first-named enlargement by tubular channels.

Gangrene in the Horse. By MM. Cadeac and Maley. *Revue Veterinaire*, Nov., 1885.—The case of gangrene following the stoppage of circulation in a limb, in consequence of arterial plugging, is not often observed. Although cases have been met with, the condition is very rare and none have been recorded in text books, so far as we have been able to ascertain. In an old horse, put aside for surgical operations, MM. Cadeac and Maley only recently noted this condition as "unique in veterinary practice." They found the lower part of the off hind limb œdematous and cold. The hair about these parts could be removed in handfuls. Autopsy showed that this gangrene of the whole of one extremity was due to the destruction of the iliac artery, external and internal of the right side, by an old blood-clot stratified and adherent, which was prolonged into all the divisions of the above arteries, without completely obstructing them, and being broken at different points. Microscopic examination showed, that the finest arterioles were plugged by the blood-clot. The inner coat of the vessels plainly showed the changes wrought by inflammation, of old-standing in some points and more recent in others. In consequence of the stagnation of blood in the vessels, hæmorrhages were produced, principally in the muscular interstices of the upper part of the limb. These authors have not been able to note the complete symptoms of this case, but, such as it is, their observation merits record in this connection.

Embolism of the Brachial Artery. Bayer. Oesterr. Vierteljahr, fur Wissensch. Veterinarkunde, 1883.—Embolism of the brachial artery shows itself by lameness on the affected side. The animal, during progression, stumbles, makes false steps, stamps constantly with the toes, or often points them in the stall and cannot flex the joints properly, but drags them stiffly along, trembling on the affected side, and occasionally falls to the ground during progression. There is no heavy breathing, no palpitation, and no swelling of any kind. After a short time the animal suddenly revives again. In a case recently under treatment of the translator, the symptoms were at first mistaken for those of cerebellar disease, as in the early stage a prominent symptom was false stepping from disordered muscular action in the limbs. Bayer recommends massage, or a kneading of the thrombus, in the treatment of these cases, through which softening and absorption follows. By methodic massage of the animal the heart muscles can be strengthened, which raises the blood pressure, the clot of blood may be removed. The animal should be rested for a few weeks, and massage, during which time has given very satisfactory results. But standing an animal too long without massage frequently aggravates the symptoms. Internally, the iodide of potassium, ergot and acetate of lead should be prescribed in full doses.

Aneurism of the Pharyngeal Artery.—M. Blaise, (*Recueil de Med.*, *Veterinaire*, 15th Aug., 1886, p. 604), veterinary surgeon in charge of the Remount Depot at Blidah, describes a case in the horse which showed swelling and induration in the region of the throat and under the maxilla, which had softened in several points, producing ulcers simulating farcy buds. There was present a frothy and profuse discharge of saliva from the mouth, having an offensive odor. Vesicants and cauterization were applied externally, and acid gargles for the mouth used, and antiseptic fumigation of the head. Under this treatment, the animal appeared to improve for a time, when, suddenly, it became worse, and died from hæmorrhage. Autopsy showed the heart, lungs and brain were healthy. Mucous membrane of the trachea showed a greenish tint; that of the pharynx was abraded and showed ulcerated points; towards the middle of the left side of the pharynx, one of the numerous ramifications of the pharyngeal artery became exposed to the length of 3 cm., by the disappearance of the mucous tissue, had dilated itself so readily, that the cellular tissues had disappeared by a slough. The pent up blood had determined an aneurism, rupture of which was inevitable. R. W. Burke, A. V. D.

Impregnation. By Dr. Fauville, Bulletin of the Society of Anthropology of Paris, 1886.

By "impregnation," Dr. Fauville means the influence which a first fecundation may exercise on those which follow, whatever the origin. In the facts, which it explains, many only see cases of atavism, of distant and unknown origin, whilst it is to be believed, on the contrary, that it is a real and general fact. In order that the "impregnation" shall be produced, four conditions are indispensible.

1st. That the fœtus presents the constitutional characteristics of the father.

2d. That the characteristics exist in its blood.

3d. That there is exchange of liquid between the mother and the fœtus.

4th. That the nourishing liquid of the mother has an influence on the ova contained in her ovaries.

The first three of these conditions are always found, and it is therefore physiologically possible that the fœtus transmits to the mother the characteristics which it receives from the father. This transmission taking place by the intermediary of the nourishing liquid, it is natural that the ova which she bears participate in the modifications undergone by the active elements which constitute her. The effect of the fœtus is the effect of the medium and nothing more. The mother will become acclimated or will succumb; but if a variation can result, it will not manifest itself on her, but on later progeny. Thus a white woman impregnated by a negro, shows no change herself; the variation will manifest itself only on the children which she will have later by a white man.

VACCINATION AND DISTEMPER IN DOGS. *Recueil de Med. Vet.*

The analogy of the cutaneous eruptions in distemper in dogs, with those of variola, was noticed by Jenner, and suggested to him the idea of vaccinating dogs to preserve them from the distemper. "It appears," he says, "that dogs are very susceptible to vaccination by inoculation, and that it produces in them all of the symptoms of distemper which is common to them, but in a foreign form, from which they do not die. They are, moreover, protected from future attacks. Out of forty-three puppies vaccinated with success none died. All were immune from contagion.

Wasbot found that distemper was inocuable, and verified the analogy with variola, but found they could contract the disease later. Dupuis, of Brussels, has just made a complete report on this subject. His experiments were made by scarification with vaccine matter, prepared at the vaccine establishment of the Crueghem School. In all dogs, young or old, whether they had had the distemper or not, the inoculation succeeded, the pustules were well formed on the eighth or ninth day, dessication commenced the eleventh or twelfth; in all, reinoculation was *without* effect, proving that the subject possessed immunity against vaccinia.

Inoculation in the veins, or by hypodermic injection, in all cases conferred immunity without producing an eruption. The vaccination has in no case proved preservative against the distemper.

The work of Dupuis establishes:

1st. That vaccinia is transmissible to the dog, and that a first inoculation protects the dog from the effects of a second.

2d. Vaccination does not protect the dog from distemper, and that distemper does not protect from vaccinia.

R. S. HUIDEKOPER, Veterinarian.

HISTORY OF A TUBERCULAR ABSCESS IN THE VERTEBRÆ COLUMN OF A Cow. By Dr. C. Verstreten, Annals et Bulletin de la Societe de Medecine de Gand, February, 1887.

Our colleague, M. Remy, lately exhibited a pathological specimen that was highly instructive as revealing certain peculiarities of Pott's disease, and the accompanying compression of the spinal cord. Heretofore, it was generally believed that the paralysis and the constrictive neuralgia which results from Pott's disease were due to a decay of the vertebræ column, and this opinion is still accepted by many writers on the subject. The fact, however, is that such paralysis and neuralgias are rather rare, for many persons are afflicted with Pott's disease who are not paralysed, nor are they troubled with constrictive pains, and in the case of the few who have suffered from these morbid conditions, both disappeared while the organic difficulty still remained. Moreover, cases are on record in which paralysis existed, the result of pressure exerted on the cord by tubercular abscesses, without any deformity of the vertebræ column itself. The fact which interests us at present is that the prominent features of Pott's disease, as it occurs in man, are identical with those in the lower animals, and that the degeneration of the nervous tissues are entirely similar. In the specimen submitted for examination, it is worth while to observe that in view of the large and numerous abscesses which existed in the vertebræ bodies, we would naturally look for an irruptional pus into the rachidian canal, and in this manner account for the compression of the spinal cord. A subsequent examination proved that such an occurrence had actually taken place. On examining more closely the specimen under consideration, we find that the cut surfaces of the vertebræ bodies contain numerous cheesy centres or foci of variable dimensions, and that these in the first state present a rose-colored appearance. If we enucleate one of the centers we bring to light a softish tumor, partly smooth, and in part covered with tufted villosities. This appearance is due to the soft covering of the tumor as lodged in the vertebræ body. This covering, about half a millimetre in thickness, adheres loosely to the adjacent bone tissue. At certain points this adherence is closer, and if we attempt to detach the fibrous from the bony wall we see long filaments of connective tissue stretching between the two which serve the purpose of feeding the tissues by imbibition. The interior of the bony receptacle is sometimes smooth and again studded with fine bony excrescences which fill up corresponding depressions in the tumor. The bone tissue which envelopes these tumors, is denser than usual, and possesses an ivory appearance. This hardened condition of the bone contributes largely to the consolidation of the diseased vertebræ, and accounts for the preservation of the normal outlines in rachitic maladies. Moreover, this unusual osseous induration occurs only where its effects are most needed, for in the case of small abscesses it is not perceived.

These large rose-colored abscesses contain a creamy substance of a certain consistency, and almost uniformly of a yellowish white or grey color and are not lobulated.

This substance is greasy to the touch, but contains small hard particles of a bony consistence, which are the result of calcification. Microscopical examination reveals beyond dispute the existence of tubercular bacilli as described by Koch. This was especially the case with the sixth cervical vertebræ. This vertebræ was filled with tubercular matter and contained six abscesses varying in diameter from 15 to 25 millimetres. One abscess existed in the transverse apophysis another extended into the trachelian prolongation of the same apophysis. A single abscess appeared in the interior of the rachidian canal, one centimetre behind the articulation of the fifth with the sixth cervical vertebræ. In all these abscesses the envel oping membrane was well defined and continuous. The seventh cervical and the three following dorsal vertebræ, present patho logical conditions which did not differ essentially from those just described as existing in the sixth cervical.

The disease as developed in the subject from which the specimen was taken, presented many peculiarities rarely met with under similar circumstances, and it is safe to say that Pott's disease is very uncommon among cows. M. Remy, superintendant of the abattoir at Gand, has assured me that he has not observed an analogous case among the different animals which daily pass under his inspection, and that he has nowhere found a similar lesion described in the annals of veterinary medicine. It is an interesting fact that the tubercular disease affected but five vertebræ, of which two were cervical and three dorsal, and this establishes a near bond of similarity to Pott's disease as it occurs in the human subject.

It seems to me there can be no doubt concerning the nature of these tumors viz.: that they are the result of osseous tuberculosis. The anatomical characters presented by the contents of the cavities hollowed out in the bodies of the cervical and dorsal vertebræ, are precisely those which occur in cheesy tubercles, however, the microscopical examination which so readily revealed the presence of Koch's bacilli, seem to me to place the matter beyond dispute. As to the question why these bacilli should infest this portion of the vertebræ column, in preference we have nothing decisive to say. It is possible that the greater nervous activity which belongs to this region may account for the presence of morbid products, which generally accompany energetic vital changes, or what is more probable, the more and abundant vascularity of those parts may account for the fact, although recent investigations continue to shed increased light on this difficult question, much yet remains to explore before a satisfactory settlement can be reached. The majority of the tubercular centers were situated within the vertebræ bodies themselves, very few extending as far as the transversal apophyses. Such at least was the case in the tumor found in the sixth cervical vertebræ. Some of the abscesses were not completely surrounded with bony tissue, but penetrated the slight bony partition which separated them from the rachidian canal. Thus the tubercular center which occupied the body of the third dorsal vertebræ, opened into the corresponding costo-vertebral articulation, and an intra-rachidian tubercle existed on the superior surface of the second dorsal vertebræ. The propagation of the tubercular center along the side of the rachidian canal fully accounts for the existence of the symptoms of fatigue which the cow in question exhibited for several days before it was slaughtered, for there must have been in consequence of such an occurence, more or less pressure of the spinal cord. Had the animal continued to live, either the tubercular abscess would have emptied into the cavity of the rachidian canal, and have given rise to an irritation of the lower surface of the *dura mater*, and a consequent pachy-meningitis or else have accompanied the course of the *dura mater* with continued increase of pressure, This explanation of the development pursued by these tumors, and their relation towards the rachidian canal, fully accounts for the symptoms observed in Pott's disease of the spine, and also explains the results which have been obtained of revulsives, setons and the actual and potential cautery. This same pressure must also be regarded as the primary cause of the constrictive pains that accompany Pott's disease.

A Case of Gastric Calculus in the Horse. By M. Remy, in the Annales et Bulletin de la Societe de Medicine de Gand.

The gastric calculus, which we have the honor of presenting, possess nearly all the characters which belong to the great variety of renal calculi observed in the horse. It has the same typical shape, being cylindrical, with the prolonged points bent in the shape of a crescent, and slightly spiral. The surface is mostly rough, as is the case with renal calculi. The convex border, however, is mostly smooth and possesses certain luminous reflection, the concave border being of a dull earthy aspect, and covered with rugosities, which present a multitude of little holes and fissures. This calculus was found in the stomach of a horse at Lille, and presents throughout three-fourths of its extent a greyish red shade, whilst the other fourth is of bluish-grey—the whole weighing 176 grammes. Gastric calculi in the horse possess a spherical shape, slightly flattened, while for the most part the surface is smooth and polished to the point of reflection, somewhat porous and slightly depressed. These calculi are composed of ammoniaco-magnesian phosphates, silicic acid, chloride of sodium, phosphate of lime, organic matter, and traces of oxide of iron. They are formed of concentric layers arranged round nucleus of foreign constitution, the latter having been conveyed into the stomach with the liquid or solid elements. These gastric calculi are rare in horses, and M. L. V. Delwart, of Cureghem, says they are so rare in monodactylous animals as to give rise to the suspicion that they do not exist among them. Such also is the testimony of other competent authorities. There exists, however, in the Veterinary School of Alfort a fragment of a gastric calculus that weighed fifty livres, but of the genuineness of this case specimen there is some doubt.

C. M. O'Leary, M. D.

